# Human keratinocyte sensitivity towards inflammatory cytokines varies with culture time

**DOI:** 10.1155/S0962935192000589

**Published:** 1992

**Authors:** G. Elliott, D. van de Meent, J. van Dijk, M. Mol

**Affiliations:** 1 Department of Pharmacology Medical Biological Laboratory T.N.O. P.O. Box 45 Rijswijk 2280 AA The Netherlands; 2 Department of Pharmacology Erasmus University P.O. Box 1738 Rotterdam 3000 DR The Netherlands

## Abstract

Proliferating keratinocyte cultures have been reported to synthesize higher concentrations of prostaglandin (PG) E than confluent ones. As interleukin-1 (IL-1) stimulates keratinocyte PGE synthesis we investigated whether the degree of confluency of the keratinocyte culture modified the response of the cells to IL-1. It was found that IL-1α (100 U/ml) stimulated PGE_2_ synthesis by proliferating (7 days in culture) but not differentiating (14 days in culture) keratinocytes. Similar effects were observed using tumour necrosis factor-α. Both arachidonic acid (AA) and the calcium ionophore A23187 stimulated PGE_2_ synthesis by 7 and 14 day cultures although the increase was greatest when 7 day cultures were used. Our data indicate that there is a specific down-regulation of the mechanism(s) by which some inflammatory cytokines stimulate keratinocyte eicosanoid synthesis as cultured keratinocytes begin to differentiate.


Mediators of Inflammation 1, 385–390 (1992)

### Human keratinocyte sensitivity towards inflammatory cytokines varies with culture time

G. Elliott,[1,CA] D. van de Meent,[1] J. van Dijk,[2] and M. Mol[1]

[1] Department of Pharmacology, Medical Biological Laboratory, T.N.O., P.O. Box 45, 2280 AA, Rijswijk, The Netherlands; [2]Department of Pharmacology, Erasmus University, Rotterdam, P.O. Box 1738, 3000 DR, Rotterdam, The Netherlands

[CA] Corresponding Author

**PROLIFERATING keratinocyte cultures have been reported to synthesize higher concentrations of prostaglandin (PG) $E_2$ than confluent ones. As interleukin-1 (IL-1) stimulates keratinocyte $PGE_2$ synthesis we investigated whether the degree of confluency of the keratinocyte culture modified the response of the cells to IL-1. It was found that IL-1α (100 U/ml) stimulated $PGE_2$ synthesis by proliferating (7 days in culture) but not differentiating (14 days in culture) keratinocytes. Similar effects were observed using tumour necrosis factor-α. Both arachidonic acid (AA) and the calcium ionophore A23187 stimulated $PGE_2$ synthesis by 7 and 14 day cultures although the increase was greatest when 7 day cultures were used. Our data indicate that there is a specific down-regulation of the mechanism(s) by which some inflammatory cytokines stimulate keratinocyte eicosanoid synthesis as cultured keratinocytes begin to differentiate.**

**Key words:** Arachidonic acid, Calcium ionophore A23187, Differentiation stage, Il-1α, Keratinocytes, $PGE_2$, TNF-α

### Introduction

Interleukin-1 (IL-1α and IL-1β) is a family of pluripotent pro-inflammatory cytokines found in high concentrations in the normal epidermis and synthesized by keratinocytes *in vitro*.[1–3] IL-1 has been reported to stimulate keratinocyte proliferation *in vitro*[4,5] and the re-epithelialization of split-skin wounds in human volunteers (personal communication). IL-1 also stimulates the synthesis of keratinocyte prostaglandin (PG) $E_2$,[6] a cyclooxygenase (CO) metabolite of arachidonic acid (AA), which enhances the proliferation of basal keratinocytes in culture and epidermal thymidine uptake *in vivo*.[7–10] It is possible, therefore, that the stimulation of keratinocyte proliferation reported for IL-1 is mediated via an increase in $PGE_2$ synthesis. However, the absence of inflammatory/proliferative reactions in the normal epidermis indicates that interactions between keratinocytes and IL-1 are tightly controlled. Keratinocyte eicosanoid synthesis varies with its state of differentiation[11,12] and proliferating keratinocyte cultures synthesize greater concentrations of $PGE_2$ than confluent ones.[7,13,14] It is possible, therefore, that sensitivity towards IL-1 is a function of the proliferative state/state of differentiation of the target cell and that proliferating and confluent keratinocyte cultures may also show differences in sensitivity towards IL-1 *in vitro*. Using cultured keratinocytes as a model system, alterations in the sensitivity of these cells towards IL-1α with time in culture were investigated, and assayed as changes in IL-1α stimulated $PGE_2$ synthesis. IL-1α was used in these experiments as it is the biologically active form of IL-1 present in the epidermis.[1] However, IL-1α and IL-1β have been shown to be equally potent in stimulating keratinocyte $PGE_2$ synthesis.[6] In order to determine the specificity of any changes observed we also monitored the effect of IL-1α on keratinocyte IL-6 and TNF-α secretion and the effect of TNF-α on keratinocyte $PGE_2$ synthesis.

### Materials and Methods

*Materials:* Antisera against $PGE_2$ were bought from Advanced Magnetics (Cambridge, MA, USA) and radiolabelled $PGE_2$ and AA from Amersham International plc (Amersham, UK). Standard $PGE_2$, AA, the calcium ionophore A23187 and actinomycin D were obtained from Sigma Chemical Company (St Louis, MO, USA). The IL-6 kits were bought from Amersham International plc (Amersham, UK) and Advanced Magnetics Inc. (Cambridge, MA, USA). Human recombinant IL-1α (HrIL-1α, activity = 10 U/ng) was a gift of S. Gillis, Immunex Research and Development Corp., Seattle, Washington, USA, and the L929 cell line was a gift of W. Fiers, State University of Ghent, Belgium. Chemicals and media for cell culture were obtained from the following companies: hydrocortisone, Sigma Chemical Co., St Louis, USA; epidermal growth factor, Gibco Laboratories, Life Technologies, New York, USA; cholera toxin, List Biological Laboratories Inc,., Campbell, CA, USA; media powders, Flow Laboratories, McClean, VA, USA.

© 1992 Rapid Communications of Oxford Ltd

*Keratinocyte culture conditions:* Keratinocytes were isolated from human breast skin, obtained from patients undergoing mammary reduction, and cultured in the presence of a 3T3 feeder layer in three parts Dulbecco's modified Eagles medium and one part Ham's F12 (DNEM/F12) supplemented with 5% foetal calf serum, 0.4 μg/ml hydrocortisone, 8.4 ng/ml cholera toxin and 10 ng/ml epidermal growth factor (complete medium) as previously described.[15] Confluent cells were trypsinized and $10^4$ cells/$cm^2$ re-seeded on a fresh feeder layer in complete medium. The day before the cultures were used, remnants of the feeder layer were removed by vigorous washing. Cultures were sometimes incubated overnight in complete medium before being used in experiments. Such secondary keratinocyte cultures usually reach confluency after 8 days. For the experiments, keratinocytes were used after 7 or 14 days in culture.

*The release of prostaglandin $E_2$, TNF-α and IL-6 from keratinocytes:* Keratinocyte cultures were incubated (37°C, 5% $CO_2$) for 3 h or 24 h[6,16] in 1 ml complete medium or DMEM/F12 with or without IL-1α. Portions of the incubation media were stored at −30°C for later analysis of $PGE_2$ and IL-6[(kit instructions)] by radioimmunoassay.[17] Analyses for TNF-α were performed immediately after the incubation period using a modification of the L929 cytotoxic assay.[18] L929 cells were cultured in complete RPMI medium (RPMI supplemented with 10% FCS, 2 mM glutamine, 100 U/ml penicillin/streptomycin and $5 \times 10^{-5}$ M mercaptoethanol). For use in the cytotoxicity assay $10^4$ viable cells (trypan blue exclusion) in 0.1 ml complete RPMI medium were transferred to 96-well plates and allowed to equilibrate overnight. The next day, 25 μl actinomycin D (final concentration 1 μg/ml) was added together with 25 μl TNF-α standard (0–500 U/ml final concentration) or 25 μl sample. The microtitre plates were then incubated overnight, the media were discarded and remaining viable cells were stained with crystal violet (0.2% in 2% ethanol) for 10 min. Microtitre plates were rinsed 5 × with tap water and the stained cells solubilized using 0.1 ml of 1% sodium lauryl sulphate. Absorbance was measured at 595 nm using a Bio-Rad model 3550 microtitre plate reader and the TNF-α activity in the samples calculated on the basis of the standard curve. Sensitivity of the assay was 0.5 U/ml TNF-α.

Cells were dissolved in sodium hydroxide and protein was measured using Bio-Rad reagent (reagent instructions). Eicosanoids, IL-6 and TNF-α are expressed in ng or units (U) per mg protein.

*The effect of IL-1α, TNF-α, arachidonic acid and A23187 on keratinocyte $PGE_2$, IL-6 and TNF-α synthesis:* For some experiments keratinocyte cultures were incubated with IL-1α (0.1–100 U/ml) or TNF-α (100 U/ml) for a 3 h period in complete medium or DMEM/F12 and the incubation media analysed for TNF-α (not TNF-α stimulated cultures). IL-6 and eicosanoids (first 3 h incubation). The keratinocytes were then washed 2 × in phosphate buffered saline and 1 ml new medium was added. The cells were then incubated for a further 3 h and the media collected for analysis of mediators (second 3 h period).[16] In other experiments cells were incubated for 24 h in complete medium or DMEM/F12 with or without IL-1α. When investigating the effects of AA (1 μg/ml) or A23187 ($10^{-6}$ M) on keratinocyte mediator synthesis the cells were incubated for 20 min in DMEM/F12.

*The effect of IL-1α on the metabolism of $^{14}C$-arachidonic acid by keratinocytes:* Day 7 keratinocyte cultures were incubated in complete medium overnight, with or without 100 U/ml IL-1α. The medium was then removed and 1 ml DMEM/F12 was added. The cultures were then incubated with [$^{14}C$]AA (0.125 μCi/ml) for a further 20 min. Radiolabelled products were extracted from the incubation medium and analyzed using a Hewlett-Packard 1084B (USA) liquid chromatograph fitted with a double pump and a temperature controlled column compartment.[17,19] Briefly, known amounts of tritiated standard eicosanoids were added to the incubation media. $^{14}C$- and $^{3}H$-labelled eicosanoids were concentrated using a SepPak $C_{18}$ cartridge (Waters Associates, USA), dissolved in ethanol and filtered through a Gelman LC3a 0.45 μm filter. Samples (100 μl) were injected onto a Zorbax $C_{18}$ HPLC column (250 × 4.6 mm i.d.; Dupont, USA). Reversed phase chromatography was performed using 30% acetonitrile/70% water (pH 2.4, 37°C) with a flow of 1 ml/min for 35 min. The acetonitrile concentration was then increased to 49% over a 13 min period and maintained at that concentration for 40 min. AA was eluted in 100% acetonitrile. Detection of radiolabelled compounds was performed using an on-line Berthold LB 506c radioactivity monitor (Wildbad, Germany) controlled by the HP 1084B terminal.

### Results

*Basal and IL-1α stimulated synthesis of eicosanoids, IL-6 and TNF-α by 7 and 14 day keratinocyte cultures:* Synthesis of $PGE_2$ during the 24 h incubation period directly following removal of the feeder layer was greater than in the subsequent 24 h period, indicating that the washing process stimulated PG formation. $PGE_2$ formation was stimulated by FCS (Table 1). The basal rate of $PGE_2$ synthesis was greater by 7 day than by 14 day keratinocyte cultures both in

**Table 1.** Prostaglandin (PG) $E_2$ synthesis by 7 day and 14 day keratinocyte cultures: the influence of (i) removing the feeder layer and (ii) foetal calf serum (FCS)

| | 7 day | | 14 day | |
|---|---|---|---|---|
| | −FCS | +FCS | −FCS | +FCS |
| First 24 h | 19.6 ± 5.8* (9) | 49.8 ± 22* (27) | 0.87 ± 0.52* (6) | 3.65 ± 0.7 (6) |
| Second 24 h | 3.63 ± 0.38 (6) | 25.25 ± 7.5 (13) | 0.69 ± 0.11 (6) | 2.81 ± 1.4 (12) |

Results, ng $PGE_2$/mg protein, are mean values ± SD. Value in brackets gives the number of replicates. Significance was calculated using the Mann–Whitney U-test. Keratinocytes were cultured on a 3T3 feeder layer in culture medium. On days 7 or 14 the feeder layer was washed away. Some cultures were then incubated for 24 h in DMEM/F12 or complete medium (first 24 h) while others were allowed to equilibrate for 24 h in complete culture medium before incubating for 24 h in DMEM/F12 or complete medium (second 24 h). * $p < 0.05$ = +FCS *vs.* −FCS and 7 day *vs.* 14 day cultures. Synthesis of $PGE_2$ during the first 24 h was higher than during the second 24 h period with the exception of 14 day cultures incubated with FCS. This effect was most marked using 7 day cultures.

the presence and absence of FCS (Table 1). In order to avoid problems associated with any nonspecific effect of washing, we used cultures 24 h after removal of the feeder layer were used. IL-1α (1–100 U/ml) stimulated the synthesis of $PGE_2$ (Fig. 1) by 7 day keratinocyte cultures during a 24 h incubation period, in the presence and absence of FCS. In contrast, IL-1α had no effect on $PGE_2$ synthesis by 14 day cultures ($PGE_2$ ng/mg protein, mean values of 4 cultures ± S.D.; DMEM/F12 control = 0.64 ± 0.14, 100 U/ml IL-1α = 0.75 ± 0.2; complete medium control = 4.18 ± 1.6, 100 U/ml IL-1α = 4.7 ± 0.9). As FCS was not essential for IL-1α stimulated PG synthesis DMEM/F12 was used in subsequent experiments. It was found that IL-1α (100 U/ml) stimulated the synthesis of $PGE_2$ by 7 day cultures during two successive 3 h incubation periods (Fig. 2). However, the stimulatory effect during the first 3 h period was not constant whereas the effect during the second period was reproducible (Table 2).

Neither IL-6 (limit of detection of assays 0.1 ng/ml, Amersham and Advanced Magnetics kits) nor TNF-α (limit of detection of bio-assay 0.5 U/ml) could be detected in the incubation medium of 7 day and 14 day cultures (incubation periods: 3 h and 24 h, with or without FCS, with or without 100 U/ml IL-1α).

*The effect of arachidonic acid and the calcium ionophore A23187 on keratinocyte prostaglandin $E_2$*: While AA and A23187 stimulated the synthesis of $PGE_2$ by both 7 and 14 day keratinocyte cultures, $PGE_2$ synthesis (ng/ml) was greater when 7 day cultures were used (Table 3). Whereas the relative stimulatory effect of AA was similar for 7 day and 14 day cultures, the relative increase in $PGE_2$ synthesis when A23187

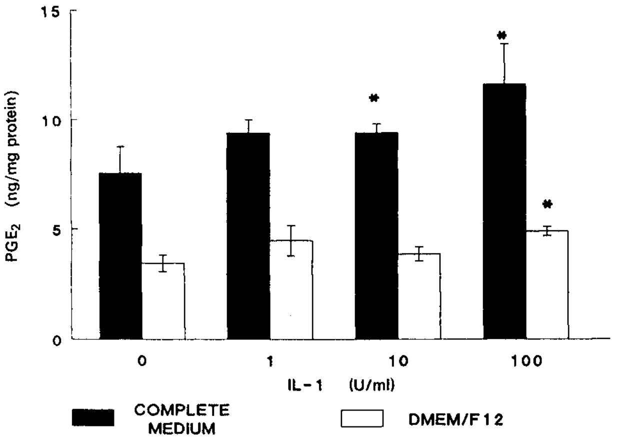

FIG. 1. The effect of interleukin-1α (IL-1α) on the synthesis of prostaglandin (PG) $E_2$ by 7 day keratinocyte cultures incubated overnight in DMEM/F12 or complete medium. Keratinocytes were cultured for 7 days and the feeder layer washed off. The cells were then cultured overnight in DMEM/F12 or complete medium with different concentrations of IL-1α. Results, ng $PGE_2$/mg protein, are mean values ± SD of triplicate incubations. Significance was calculated using the Mann-Whitney U-test. * $p = < 0.05$ *vs.* corresponding control.

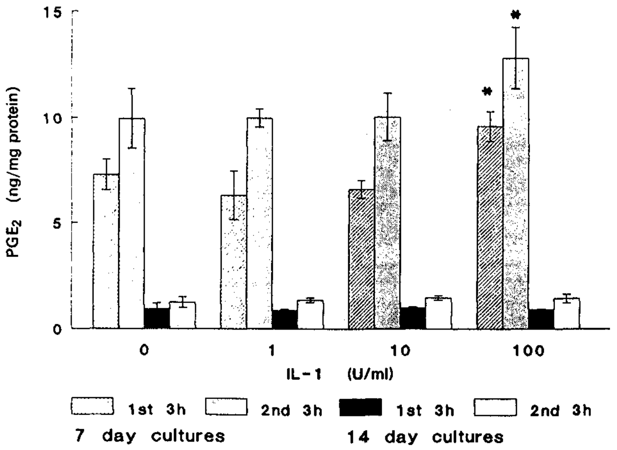

FIG. 2. The effect of interleukin-1α (IL-1α) on prostaglandin (PG) $E_2$ synthesis by 7 and 14 day keratinocyte cultures during consecutive 3 h incubation periods. Keratinocytes were cultured for 7 or 14 days and the feeder layer washed off. The cells were then cultured for 3 h in DMEM/F12 or complete medium with different concentrations of IL-1α (first 3 h). The medium was then removed and replaced with fresh medium for a further 3 h (second 3 h). Results, ng $PGE_2$/mg protein, are mean values ± SD of triplicate incubations. Significance was calculated using the Mann-Whitney U-test. * $p = < 0.05$ *vs.* corresponding control.

**Table 2.** The effect of preincubating 7 and 14 day keratinocyte cultures with IL-1α on the subsequent synthesis of prostaglandin (PG) $E_2$

| | Day 7 cultures | | Day 14 cultures | |
|---|---|---|---|---|
| | First 3 h | Second 3 h | First 3 h | Second 3 h |
| Expt. 1 | | | | |
| control | 4.64 ± 0.84 | 17.94 ± 1.1 | 0.36 ± 0.11 | 0.88 ± 0.14 |
| +IL-1α | 5.86 ± 0.22* | 22.6 ± 1.9* | 0.44 ± 0.04 | 0.87 ± 0.26 |
| Expt. 2 | | | | |
| control | 8.28 ± 0.72 | 9.96 ± 1.3 | 1.88 ± 0.28 | 2.52 ± 0.50 |
| +IL-1α | 9.52 ± 0.72 | 12.76 ± 1.2* | 1.84 ± 0.08 | 3.66 ± 1.28 |
| Expt. 3 | | | | |
| control | 5.2 ± 1.6 | 7.4 ± 1.4 | N/D | N/D |
| +IL-1α | 7.8 ± 0.2* | 20.0 ± 1.0* | N/D | N/D |

Results, ng $PGE_2$/mg protein, are mean values ± SD of triplicate incubations. Significance was calculated using the Mann–Whitney U-test. * $p < 0.05$ *vs.* corresponding control. Keratinocytes were cultured for 7 or 14 days. The feeder layer was then removed and the cells allowed to equilibrate overnight. They were then incubated for 3 h in DMEM/F12, with or without 100 U/ml IL-1α (first 3 h). The cultures were then washed with DMEM/F12 and incubated for a second, consecutive 3 h period without IL-1α (second 3 h). After each 3 h the incubation media were analysed of $PGE_2$. 14 day values were significantly lower than 7 day values.

was incubated with 7 day cultures was greater than when 14 day cultures were used (Table 3).

*The effect of IL-1α on the metabolism of radiolabelled arachidonic acid by 7 and 14 day keratinocyte cultures:* Radiolabelled AA was metabolized by 7 day keratinocyte cultures to a variety of CO and lipoxygenase metabolites. Conversion of AA, during the 20 min incubation, was small and less than 1% of added radioactivity was associated with metabolites recovered from the incubation medium. Ther was some variation between replicates and

**Table 3.** Arachidonic acid (AA) and calcium ionophore (A23187) stimulate prostaglandin (PG) $E_2$ synthesis by 7 day and 14 day old keratinocyte cultures

| Treatment | Time in culture | | | |
|---|---|---|---|---|
| | 7 days | | 14 days | |
| | Expt. 1 | Expt. 2 | Expt. 1 | Expt. 2 |
| Control | 1.30 ±0.20 | 1.86 ±0.37 | 0.69 ±0.11 | 0.23 ±0.02 |
| AA | 29.66 ±2.70* (22.82) | 21.70 ±6.00* (11.67) | 13.67 ±1.38* (19.80) | 4.12 ±1.33* (17.91) |
| Control | 2.52 ±0.74 | 2.22 ±0.60 | 0.94 ±0.07 | 0.25 ±0.03 |
| A23187 | 11.34 ±0.50* (4.50) | 7.44 ±1.2* (3.35) | 1.63 ±0.18* (1.73) | 0.73 ±0.20* (2.92) |

Results, ng $PGE_2$/mg protein, are mean values ± SD of triplicate incubations. Significance was calculated using the Mann–Whitney U-test. * $p < 0.05$ *vs.* corresponding control. Values in brackets give the relative increases in $PGE_2$ synthesis due to AA and A23187. Keratinocytes were cultured for 7 or 14 days and the feeder layer washed off. The cells were then cultured overnight in complete medium before incubating them in DMEM/F12 for 20 min, with or without AA or A23187.

only metabolites detected in three separate experiments are mentioned. Eicosanoids co-eluting with $^3H$-$PGD_2$, $PGE_2$, 6-keto-$PGF_{1\alpha}$, 15-hydroxy-eicosatetraenoic acid (15-HETE), leukotriene (LT) $C_4$, $LTB_4$ and a non-enzymic hydrolysis product of $LTB_4$, epi-$LTB_4$, were detected. An HPLC trace showing the positions of [$^3H$]- and [$^{14}C$]-eicosanoids is given in Figure 3. Preincubating 7 day keratinocyte cultures with IL-1α (1–100 U/ml) resulted in a small increase in the synthesis of radiolabelled $PGE_2$ in those cultures incubated with 100 U/ml (mean dpm ± S.D., n = 3, control = 320 ± 10, IL-1α 100 U/ml = 560 ± 50; % of total metabolite dpm, control = 23% ± 4.0, IL-1α 100 U/ml = 34% ± 6.0). There were no significant increases in the synthesis of other eicosanoids.

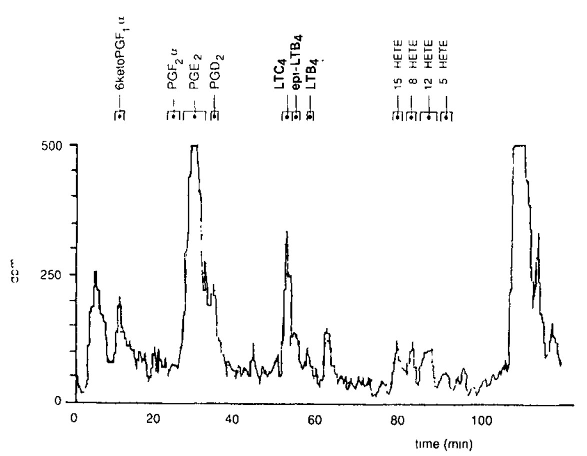

FIG. 3. Reversed phase HPLC separation of eicosanoids produced by adherent human skin keratinocytes after incubation with radiolabelled arachidonic acid. Retention times of non-labelled and labelled standard eicosanoids are indicated above the trace (·). ⊓ = Peak width of both [$^{14}C$] labelled metabolites and [$^3H$] labelled standards used to calculate eicosanoid dpm.

**Table 4.** The effect of tumour necrosis factor alpha (TNF-$\alpha$) on keratinocyte prostaglandin (PG) $E_2$ synthesis by 7 and 14 day old keratinocyte cultures

| | 7 day | 14 day |
|---|---|---|
| Control | 7.18 ± 1.02 | 4.24 ± 1.13 |
| TNF-$\alpha$ (100 U/ml) | 17.54 ± 2.48* | 4.83 ± 1.04 |

Results, ng PGE/mg protein, are mean values ± SD ($n = 3$). Significance was calculated using the Mann-Whitney U-test. * $p < 0.05$ *vs.* corresponding control. Keratinocytes were cultured for 7 or 14 days. The feeder layer was then removed and the cells allowed to equilibrate overnight. They were then incubated for 3 h in DMEM/F12 with or without 100 U/ml TNF-$\alpha$.

*The effect of TNF-$\alpha$ on keratinocyte eicosanoid synthesis:* TNF-$\alpha$ (100 U/ml) stimulated the synthesis of $PGE_2$ by 7 day keratinocyte cultures during a 3 h incubation. It had no effect on PG formation by 14 day cultures (Table 4).

### Discussion

The sensitivity of human keratinocytes towards IL-1$\alpha$ and TNF-$\alpha$ *in vitro* varied with time in culture. It was found that IL-1$\alpha$ and TNF-$\alpha$ stimulated $PGE_2$ synthesis by keratinocytes after 7 days, but not 14 days, in culture. This loss of sensitivity towards IL-1$\alpha$ and TNF-$\alpha$ was associated with a reduction in both basal and stimulated (AA and A23187) $PGE_2$ formation. Although $PGE_2$ synthesis was enhanced when foetal calf serum was added to the incubation medium, maybe due to the presence of AA in the serum, IL-1$\alpha$ and TNF-$\alpha$ stimulation of $PGE_2$ synthesis was not dependent on the presence of serum or added growth factors. The finding that pre-exposure of keratinocytes to IL-1$\alpha$ was sufficient to stimulate $PGE_2$ synthesis (first and second 3 h incubations) is consistent with reports that IL-1$\alpha$ stimulated AA turnover in fibroblasts and keratinocytes is dependent on *de novo* protein synthesis.[6,16,20-22] Data obtained using radiolabelled AA are in general agreement with reports indicating that keratinocytes are capable of synthesizing different cyclooxygenase (CO) ($PGD_2$, 6-keto $PGF_{1\alpha}$, $PGE_2$) and lipoxygenase (15-HETE, $LTB_4$ and leukotriene $C_4$) metabolites of AA.[11-13,23-25] However, it appears from our data that the stimulatory action of IL-1$\alpha$ could be selective for $PGE_2$ although this has to be confirmed using RIAs. AA stimulated $PGE_2$ synthesis by 7 day keratinocyte cultures, calculated as the relative increase, was the same as for 14 day cells. The differences in AA stimulated $PGE_2$ formation by 7 and 14 day cultures can, therefore, be ascribed to changes in keratinocyte CO concentrations. The stimulatory effect of A23187 on 7 day cultures was, in contrast, greater in both absolute (per mg protein) and relative terms than its effect on 14 day cultures. This finding suggests that intra-cellular, $Ca^{2+}$ sensitive, mechanisms controlling the level of free AA are up- or down-regulated depending on the degree of differentiation of the culture. Increases in *de novo* CO and phospholipase $A_2$, a $Ca^{2+}$ sensitive enzyme which releases AA from membrane phospholipid stores,[20] have been shown to occur in fibroblasts after exposure to IL-1.[20-22] It is possible therefore that an increase in the synthesis of these enzymes is coupled to those mechanisms regulating keratinocyte proliferation/differentiation. As IL-1$\alpha$ and TNF-$\alpha$ were without effect on 14 day cultures it appears that there are also specific alterations in the receptor mediated signal transduction system by which these cytokines stimulate $PGE_2$ formation. Our data are consistent with the concept that, as keratinocyte cultures proliferate and become confluent, there are changes in basal and stimulated $PGE_2$ synthesis which can be attributed to alterations at receptor, second messenger and CO levels.

The present results support previous publications showing that non-confluent keratinocyte cultures release higher concentrations of $PGE_2$ than confluent ones.[7,13,14] However data obtained using different isolated epidermal cell populations is contradictory and authors have reported that both differentiated and basal keratinocytes are the most active in metabolizing AA.[11,12] These differences have yet to be reconciled. Concentrations of IL-6 and TNF in keratinocyte incubation media were below the levels of detection of the assays used, for all the experiments performed. This finding is in contrast to other publications reporting that IL-6 release from keratinocytes is enhanced by IL-1.[3,26] Those authors assayed IL-6 using the B9 cell bioassay however and we are not aware of any report where the release of IL-6 from keratinocytes was measured by RIA. It is possible that the different assay systems do not yield compatible results, although IL-6 secretion by human fibroblast was easily detected using the Amersham kit.[16] In view of the lack of effect on secretion of IL-6 or TNF-$\alpha$, it is unlikely that the stimulatory effect of IL-1$\alpha$ on keratinocyte $PGE_2$ synthesis was mediated via a stimulation of secondary cytokines.

In summary, it has been shown that there are alterations in the sensitivity of keratinocytes towards IL-1$\alpha$ and TNF-$\alpha$ as keratinocyte cultures become confluent and begin to differentiate. The down-regulation of $PGE_2$ synthesis observed, as keratinocytes begin to differentiate, occurs at the receptor, second messenger and CO levels. Such changes in the sensitivity of keratinocytes towards inflammatory cytokines could be an important mechanism by which the response of the epidermis to injury (re-epithelialization) or noxious stimuli (hyperplasia) is regulated.

### References

1. Schmitt A, Hauser C, Jaunin F, Dayer JM, Saurat JH. Normal epidermis contains high amounts of natural tissue IL-1. Biochemical analysis by HPLC identifies a MW 17 KDa form with a Pi 5.7 and a MW 30 kDa form. *Lymphokine Res* 1986; **5**: 105–118.
2. Kupper T, Ballard DW, Chua AO, *et al.* Human keratinocytes contain mRNA indistinguishable from monocyte interleukin 1α and 1β mRNA. *J Exp Med* 1986; **164**: 2095–2100.
3. Partridge M, Chantry D, Turner M, Feldman M. Production of interleukin-1 and interleukin-6 by human keratinocytes and squamous cell carcinoma cell lines. *J Invest Dermatol* 1991; **96**: 771–776.
4. Ristow HJ. A major factor contributing to epidermal proliferation in inflammatory skin diseases appears to be interleukin-1 or a related protein. *Proc Natl Acad Sci USA* 1987; **84**: 1940–1944.
5. Sauder DN. Biological properties of epidermal thymocyte-activating factor (ETAF). *J Invest Dermatol* 1985; **85**: 176S–182S.
6. Pentland AP, Mahoney MG. Keratinocyte prostaglandin synthesis is enhanced by IL-1. *J Invest Dermatol* 1990; **94**: 43–46.
7. Pentland AP, Needleman P. Modulation of keratinocyte proliferation *in vitro* by endogenous prostaglandin synthesis. *J Clin Invest* 1986; **77**: 246–251.
8. Bem JL, Greaves MW. Prostaglandin $E_1$ effects on epidermal cell growth *in vitro*. *Arch Dermatol Forsch* 1974; **251**: 35–41.
9. Furstenberger G, Marks F. Indomethacin inhibition of cell proliferation induced by the phorbol ester TPA is reversed by prostaglandin E2 in mouse epidermis *in vivo*. *Biochem Biophys Res Commun* 1978; **84**: 1103–1111.
10. Eaglstein WH, Weinstein GD. Prostaglandin and DNA synthesis in human skin: possible relationship to ultraviolet light effects. *J Invest Dermatol* 1975; **64**: 386–389.
11. van Zepelin HHH, Schröder J, Smid P, Reusch MK, Christophers E. Metabolism of arachidonic acid by human epidermal cells depends on maturation stage. *J Invest Dermatol* 1991; **97**: 291–297.
12. Cammeron GS, Baldwin JK, Jasheway DW, Pattrick KE, Fisher SM. Arachidonic acid metabolism varies with the state of differentiation in density gradient-separated mouse epidermal cells. *J Invest Dermatol* 1990; **94**: 292–296.
13. Rice H, Levine L. Melittin-stimulated arachidonic acid metabolism by cultured malignant epidermal keratinocytes. *Biochem Biophys Res Comm* 1984; **124**: 303–307.
14. Pentland AP, George J, Moran C, Needleman P. Cellular confluence determines injury-induced prostaglandin E2 synthesis by human keratinocyte cultures. *Biochem Biophys Acta* 1987; **919**: 71–78.
15. Mol MAE, van de Ruit BC, Kluivers AW. $NAD^+$ levels and glucose uptake of cultured epidermal cells exposed to sulfur mustard. *Toxicol Appl Pharmacol* 1989; **98**: 150–165.
16. Elliott G, van de Meent D, van Dijk J. Interleukin-1α-stimulated fibroblast eicosanoid synthesis is not mediated by interleukin-6. *Eur J Pharmacol* 1992; **214**: 253–259.
17. Zijlstra FJ, Vincent JE. Determination of leukotrienes and prostaglandins in [$^{14}$C] arachidonic acid labelled human lung tissue by high-performance liquid chromatography and radioimmunoassay. *J Chromatography* 1984; **311**: 39–50.
18. Ruff MR, Gifford GE. Tumor necrosis factor. In: Pick E, ed. *Lymphokines 2*. New York: Academic Press, 1981; 235.
19. Van der Ham AC, Kort WL, Zijlstra FJ, Vermeer MA, Jeekel J. Eicosanoid profile of healing colon anastomosis and peritoneal macrophages in the rat. *Gut* 1989; **31**: 807–811.
20. Godfrey RW, Johnson WJ, Hoffstein ST. Interleukin-1 stimulation of phospholipase activity in rat synovial fibroblasts. *Arthritis Rheum* 1988; **31**: 1421–1428.
21. Korn JH, Downie E, Roth GJ, Ho SY. Synergy of interleukin 1 (IL-1) with arachidonic acid and A23187 in stimulating prostaglandin E synthesis in human fibroblasts: IL-1 stimulates fibroblast cyclooxygenase. *Clin Immunol Immunopathol* 1989; **50**: 196–204.
22. Raz A, Wyche A, Siegel N, Needleman P. Regulation of fibroblast cyclooxygenase synthesis by interleukin-1. *J Biological Chem* 1988; **263**: 3022–3028.
23. Kvedar J, Levine L. Modulation of arachidonic acid metabolism in a cultured newborn rat keratinocyte cell line. *J Invest Dermatol* 1987; **88**: 124–129.
24. Kondoh H, Sato Y, Kanoh H. Arachidonic acid metabolism in cultured mouse keratinocytes. *J Invest Dermatol* 1985; **85**: 64–69.
25. Rosenbach T, Czernielewski J, Hecker M, Czarnetzki B. Comparison of eicosanoid generation by highly purified human Langerhans cells and keratinocytes. *J Invest Dermatol* 1990; **95**: 104–107.
26. Kirnbauer R, Kock A, Schwarz T, *et al.* IFN-β2, B cell differentiation factor 2, or hybridoma growth factor (IL-6) is expressed and released by human epidermal cells and epidermoid carcinoma cell lines. *J Immunol* 1989; **142**: 1922–1928.

ACKNOWLEDGEMENTS. We are pleased to acknowledge the excellent technical assistance of Anne-Marij de Vries who cultured the keratinocytes.

**Received 1 September 1992; accepted in revised form 16 September 1992**
